# Laser‐Induced Heterostructuring of Graphene Passivated Nanoscale Black Phosphorus Frameworks for Lithium‐Ion Battery Anodes

**DOI:** 10.1002/smll.202504480

**Published:** 2025-09-01

**Authors:** Sujit Deshmukh, Pawel Jakobczyk, Krzysztof Pyrchla, Maria Brzhezinskaya, Mateusz Ficek, Bing Yang, Nianjun Yang, Robert Bogdanowicz

**Affiliations:** ^1^ Faculty of Electronics Telecommunications, and Informatics Gdansk University of Technology 11/12 G. Narutowicza Str. Gdansk 80‐233 Poland; ^2^ Helmholtz‐Zentrum Berlin für Materialien und Energie Hahn‐Meitner‐Platz 1 14109 Berlin Germany; ^3^ Shenyang National Laboratory for Materials Science Institute of Metal Research (IMR) Chinese Academy of Sciences (CAS) No. 72 Wenhua Road Shenyang 110016 China; ^4^ Department of Chemistry Hasselt University Agoralaan‐Gebouw F Wetenschapstoren Kantoor F4.12 Diepenbeek 3590 Belgium; ^5^ Institute of Materials Research Hasselt University Wetenschapspark 1 Diepenbeek 3590 Belgium

**Keywords:** *ab initio* modelling, black phosphorus, lithium‐ion battery, sp^2^ carbon frameworks, X‐ray spectroscopy

## Abstract

Two‐dimensional black phosphorus (BP or phosphorene) has drawn significant interest in alkali metal ion storage due to its capacity to adsorb alkali atoms and high theoretical prediction of specific capacity. But the problem persists in large‐scale production of the nanoscale BP, low electronic conductivity, considerable volume change (≈300%), and polyphosphide‐induced shuttle effect. To solve this problem, a single‐step lasing method is employed to prepare nanoscale BP covalently bound to the sp2 bonded carbon framework through a P─O─C/P─C bond. The sp2 bonded carbon provides exceptional electrical conductivity, while BP offers high theoretical capacity. The possible bond formation between carbon, oxygen, and phosphorus atoms was studied using synchrotron‐based X‐ray photoelectron spectroscopy and near‐edge X‐ray absorption fine structure spectroscopy. The experimental findings were supported by the ab‐initio density functional theory modelling and REAX FF molecular dynamics simulations. By adopting such structure, an ultrastable lithium‐ion battery (LIB) cell was developed with ≈100 % coulombic efficiency till 700 cycles at 2 A g^−1^ current density. Theoretical computation reveals that interlayer covalent bonding is a crucial mechanism for this stable device performance during Li^+^ intercalation/deintercalation process. This study provides valuable insights into the customized fabrication of nanoscale 2D heterostructure using laser techniques, focusing on long‐lasting LIBs.

## Graphical abstract

Nanoscale BP‐LIG anode for LIB! The work demonstrates a simple laser method to fabricate a BP‐LIG hybrid anode by decorating nanoscale BP particles onto a 3D porous LIG network. Simple laser processing enables *in situ* anchoring of nanoscale BP on the 3D network of LIG via P─C covalent bonding. The resulting electrode delivers a capacity of 1122 mAh g^−1^ with 91% capacity retention after 400 cycles.

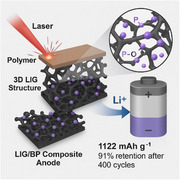

## 1 Introduction

Delamination and exfoliation of black phosphorus (BP) into single/multilayer or preparation of nanoscale thin BP with a wide range of promising applications that are unachievable in bulk structure have recently garnered significant interest. BP offers a tunable direct band gap (0.3 eV (bulk) to 2 eV (monolayer)), positioning it between wide‐band‐gap transition metal dichalcogenides (TMDs) and semi‐metallic graphene.^[^ ^1^, ^2^, ^3^ ^]^ Additionally, it has high carrier mobility (>800 cm^2^ V^−1 ^s^−1^) and in‐plane anisotropic properties, making it highly suitable for various electronics and optoelectronics applications.^[^ ^4^, ^5^, ^6^, ^7^, ^8^ ^]^ These include transistors, infrared photodetectors, modulators, plasmonic devices, etc.^[^ ^8^, ^9^, ^10^, ^11^ ^]^ As a 2D analogue of graphene, BP can be exfoliated into few‐layer forms by breaking its weak interlayer van der Waals interactions, which has the potential to replace graphene as anode materials for alkali ion batteries (Li/Na/K) especially lithium‐ion batteries (LIBs) which is one of the dominant power sources for wearable and portable electronic devices.^[^ ^12^, ^13^ ^]^ Note that the theoretical study supports that the Li diffusion along the zigzag path of phosphorene is 10^2^ orders of magnitude higher than MoS_2_ and graphene.^[^ ^14^, ^15^, ^16^, ^17^ ^]^ As an anode material for LIB, the theoretical capacity of BP is predicted as 2596 mAh g^−1^, which is seven times higher than the commonly used commercial graphite (372 mAh g^−1^).^[^ ^18^, ^19^, ^20^ ^]^ Nevertheless, the challenge lies in the rapid capacity fading caused by the huge volumetric change (≈300 %) during Li‐ion intercalation cycling and polyphosphide‐induced shuttle effects.^[^ ^20^, ^21^, ^22^ ^]^ Another problem is the formation of the Li_3_P phase during the cycling process, which destroys the crystal structure of BP.^[^ ^23^, ^24^ ^]^ To prolong the cyclic stability, preparing nano‐dimension BP attached with a high conductive network could be an effective way to accommodate the large volume expansion and shorten the ion diffusion path due to the conductive support network.

In this direction, progress has been achieved by preparing phosphorene‐graphene composite anodes for sodium and potassium ion batteries.^[^ ^12^ ^]^ In these hybrid structures, graphene layers served as a mechanical backbone and an electrical conduction highway, compensating for the lower electrical conductivity of phosphorene. Apart from the nanocarbon addition, Gue et al. presented metal‐ion‐modified BP with enhanced stability.^[^ ^25^ ^]^ Wang et al. reported, p‐Toluenesulfonyl isocyanate as an electrolyte additive to overcome the problem of electrolyte decomposition and solid electrolyte interface crack.^[^ ^26^ ^]^ Ma et al. reported the covalent bonding between metallic antimony and BP to ensure fast Li^+^ transport.^[^ ^21^ ^]^ The integration of BP with a conductive carbon network is still the most common and facile approach to improve the electrochemical properties of BP.^[^ ^27^, ^28^ ^]^ However, there are still some bottlenecks in the preparation of phosphorus‐carbon composite. This synthesis involves the liquid phase exfoliation either by extensive sonication or mixing, but the strong interlayer interactions present a challenge to the exfoliation process.^[^ ^29^, ^30^, ^31^ ^]^ There are two commonly used solvents used for the exfoliating agent, such as *N* ‐methyl‐2‐pyrrolidone (NMP) and dimethylformamide (DMF), but further research is needed to identify suitable solvents with surface energies that better match that of BP for more efficient exfoliation. Additionally, the primary challenge for practical applications has been the absence of large‐scale production methods of nano‐dimension BP particles and the difficulty in organizing these properties into a flexible structured architecture for portable electronics.

Recently, an alternate chemical‐free route for the microfabrication of carbon‐based material on flexible substrate received great attention due to its ease of cost‐effective roll‐to‐roll production.^[^ ^31^, ^32^, ^33^ ^]^ The process uses direct laser scribing on flexible polymers (polyimide, polyesters, etc.), which can be photothermally converted to a range of conductive carbon materials. A common example of such carbon material is laser‐induced graphene (LIG). Laser‐based carbon materials are characterized by high porosity, high electrochemical stability in a wide range of electrolytes, good electrical conductivity, and tunable microstructure due to their multidoping nature.^[^ ^31^, ^32^, ^33^ ^]^ These properties align with the key requirements for an ideal current collector. Liu et al. reported that, the lower defect level the carbon led to a more stable Li plating‐stripping cycle.^[^ ^34^ ^]^ In contrast, the defective carbon may lead to thick SEI formation, which will promote Li metal dendrite growth.^[^ ^34^ ^]^ Yang et al. recently reported stable cycling of lithium‐metal anodes when LIG was introduced as a current collector, in which LIG's dopant sites assisted the lithium nucleation kinetics.^[^ ^35^ ^]^

Here in this work, we reported a chemical‐free facile process to fabricate carbon‐supported BP particles functionalized and linked with a sp^2^ framework of graphene through P─C bonding, named BP‐LIG. The process involves a single‐step lasing on a BP‐coated flexible polymer (polyimide) surface. The BP particles absorb the laser energy and fragment into nano dimensions. At the same time, polyimide sheet is photothermally converted into a porous 3D graphene structure with the release of carbonized steam. A detailed material characterization, including structural, morphological, electronic structure, and chemical bonding analysis, together with electrochemical analyses, is presented to clarify the fundamental properties of the 2D BP‐LIG hybrid architecture. The formation of P─C and P─O─C covalent bonds creates a stable structure and ensures robust contact between BP and the LIG matrix. Thanks to this stability, the BP‐LIG electrode achieved a specific capacity of 1122 mAh g^−1^ at 0.5 A g^−1^ and retained 91% of its capacity after 400 cycles, even at a high current density of 2 A g^−1^. Theoretical studies offer valuable insights regarding the bond formation between the BP and LIG framework and electrochemical processes of the BP‐LIG structure during Li intercalation. This work lays a solid foundation for advancing laser‐based processing techniques for nanoscale 2D BP materials in a wide range of energy storage applications.

## 2 Results and Discussions

### 2.1 Synthesis and Characterization of BP‐LIG Hybrid

**Figure 1:**
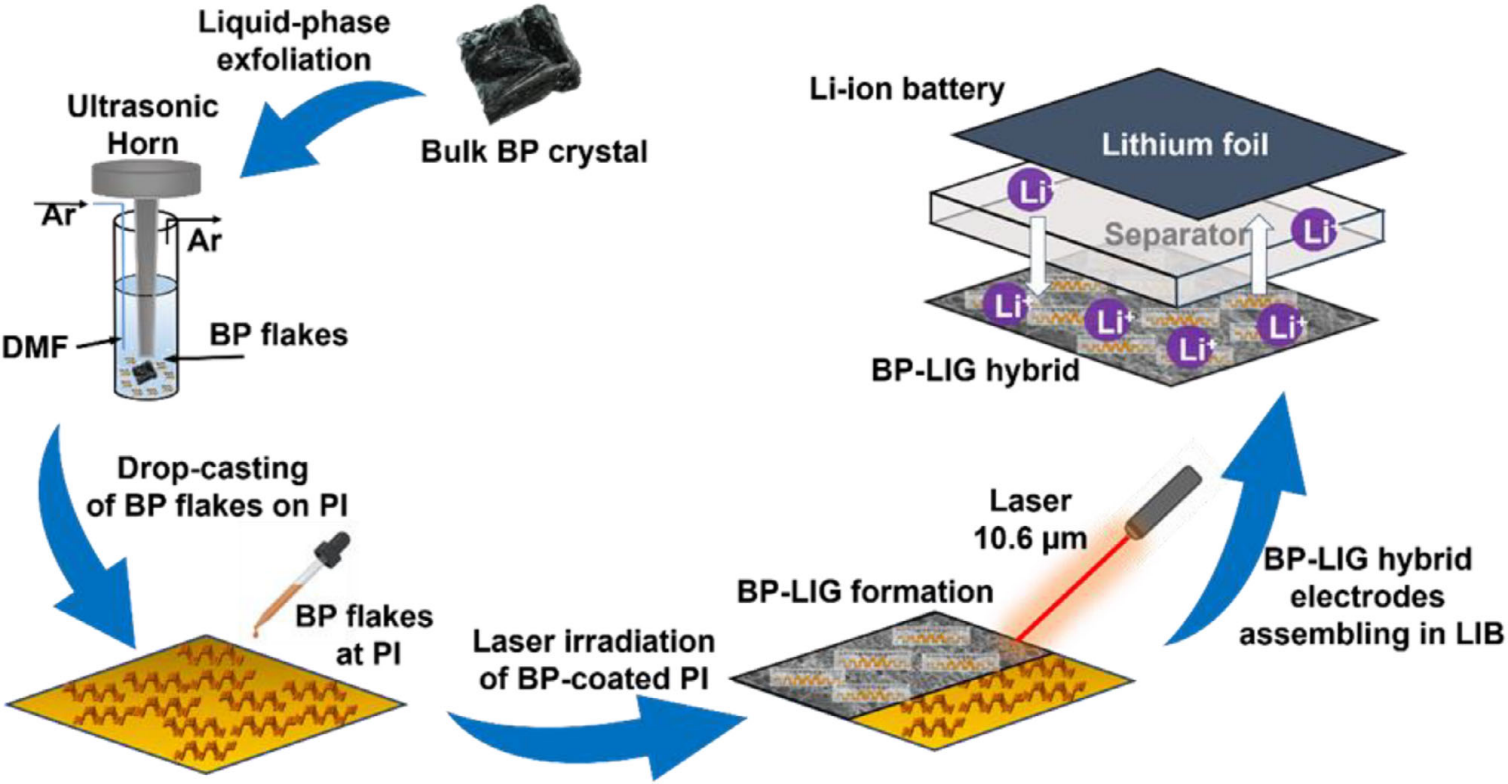
Schematic diagram of the workflow. Laser‐based synthesis of BP‐LIG hybrid structure and fabrication of LIB device.

A schematic illustration for the fabrication of BP‐LIG hybrid using a single‐step lasing method is shown in **Figure** **1**. 1st few‐layer black phosphorus (FLBP) was prepared by solvent‐assisted exfoliation, and then uniform dispersion of 2D BP sheets was coated on a flexible polyimide film, followed by the direct laser (CO_2_ laser; 10.6 µm) rastering on the BP‐coated polyimide film. During the laser rastering process, the laser power was maintained at a constant 4 W, while the scan rate was set to 150 mm s^−1^. The infrared laser energy generates sufficient mechanical stress on the BP particles, breaking the 2D sheets into nanometric dimensions. At the same time, polyimide film released carbonized steam by absorbing the laser energy and transforming it into a porous 3D network of LIG. This straightforward laser method, therefore, enables the *in situ* decoration of nanoscale BP particles onto a 3D LIG network. The BP‐LIG hybrid was finally assembled in LIB configuration.

**Figure 2:**
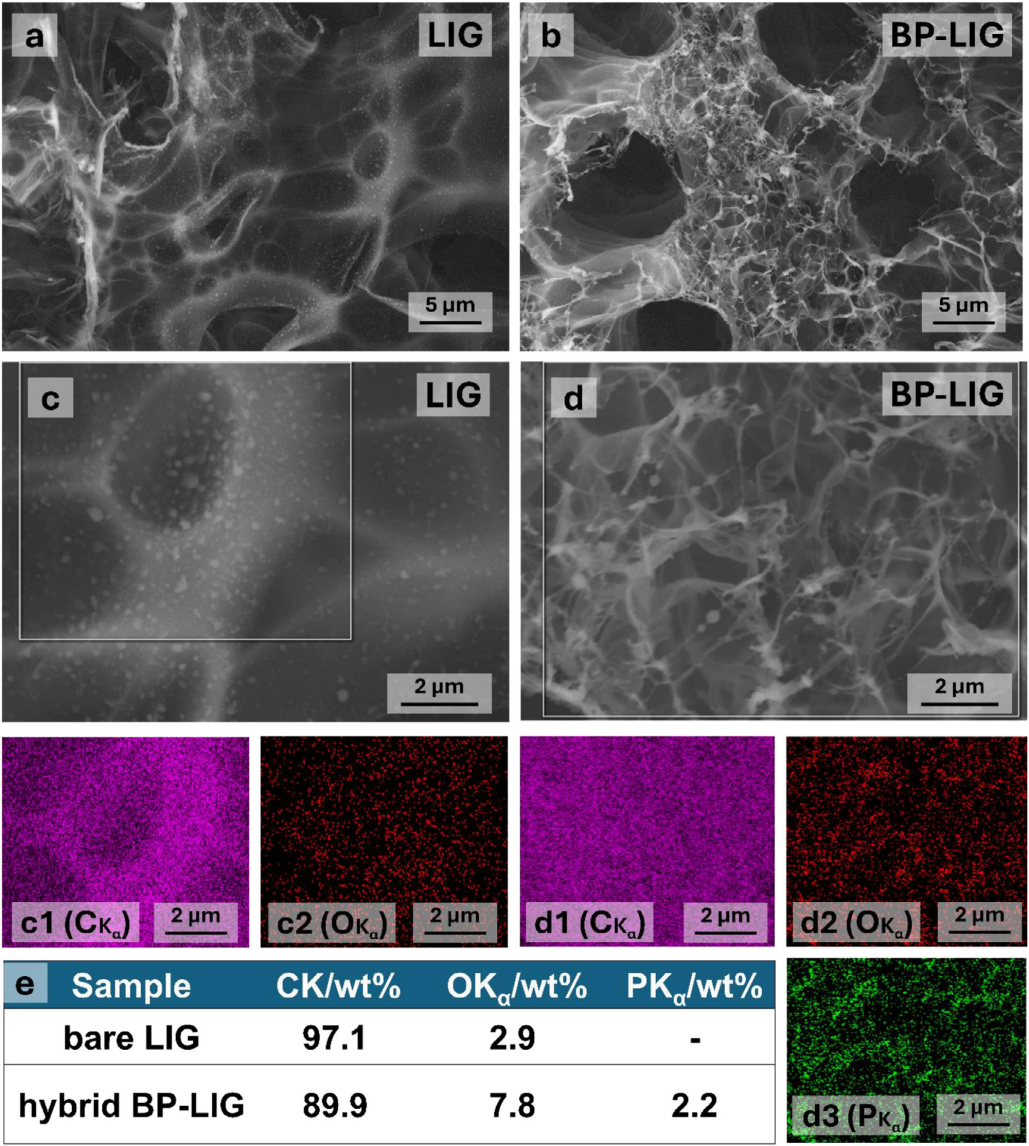
Morphological characterizations. SEM top view of a) LIG and b) BP‐LIG hybrid. EDX elemental mapping of c,c1,c2) LIG and d,d1,d2,d3) BP‐LIG hybrid, revealing the presence of C, O, and P. **e)** Extracted elemental distribution in tabulated form. The results were extracted from EDX spectra.

The successful attachment of BP particles on the LIG surface was confirmed by scanning electron microscopy (SEM) analysis. **Figure** **2**a illustrates that LIG films display a sheet‐like morphology with a 3D porous structure, formed by the rapid release of carbonaceous gases from flexible polyimide film.^[^ ^36^ ^]^ Figure 2b illustrates the uniform distribution of BP particles over the LIG network. Note that the BP‐LIG hybrid appears to be more porous as compared to LIG. This type of structure, featuring the distribution of nano BP particles bonded to graphene, not only enhances space utilization but also effectively prevents the self‐restacking of both 2D BP and graphene sheets.

To further confirm the uniform distribution of BP particles throughout the LIG framework, additional elemental mapping analysis was conducted. Energy‐dispersive X‐ray spectroscopy (EDX) mapping confirms the presence of carbon (C) and oxygen (O) in LIG (Figure 2c,c1,c2), while the BP‐LIG hybrid shows a uniform distribution of phosphorus (P) alongside C and O (Figure 2d,d1,d2,d3). The corresponding EDX spectra of LIG and BP‐LIG are displayed in Figures S1 and S2 (Supporting Information). Figure 2e represents the atomic percentage of elements (extracted from EDX spectra) present in LIG and BP‐LIG. Note that O at% increased 3 times in BP‐LIG as compared to LIG. This increase results from laser‐induced high‐temperature thermal oxidation of BP at the nanoscale dimension, similar to observations previously reported on the surface of 2D MXene.^[^ ^37^ ^]^

A typical HRTEM image of the BP‐LIG sample is displayed in Figure S3 (Supporting Information). The characteristic wrinkles of laser‐processed graphitic carbon are visible as reported previously.^[^ ^33^, ^38^ ^]^ The laser‐induced compressive strain creates the characteristic wrinkles on LIG. The few‐layer structure is evidenced on the BP‐LIG surface. The interlayer spacing of LIG was calculated as ≈0.38 nm, which is slightly wider compared to graphitic (002) carbon. The wider interlayer spacing is associated with the attached oxygenated functional group on the BP‐LIG surface.^[^ ^39^ ^]^

To expand the information on the possible formation of chemical bonds between carbon, oxygen, and phosphorus atoms in BP‐LIG, Raman, X‐ray photoelectron spectroscopy (XPS), and near‐edge X‐ray absorption fine structure (NEXAFS) spectroscopy methods were also used in this investigation. It is worth mentioning that stable covalent bond formation between BP and carbon atoms is known to improve the electrochemical performance of LIB.^[^ ^23^ ^]^

**Figure 3:**
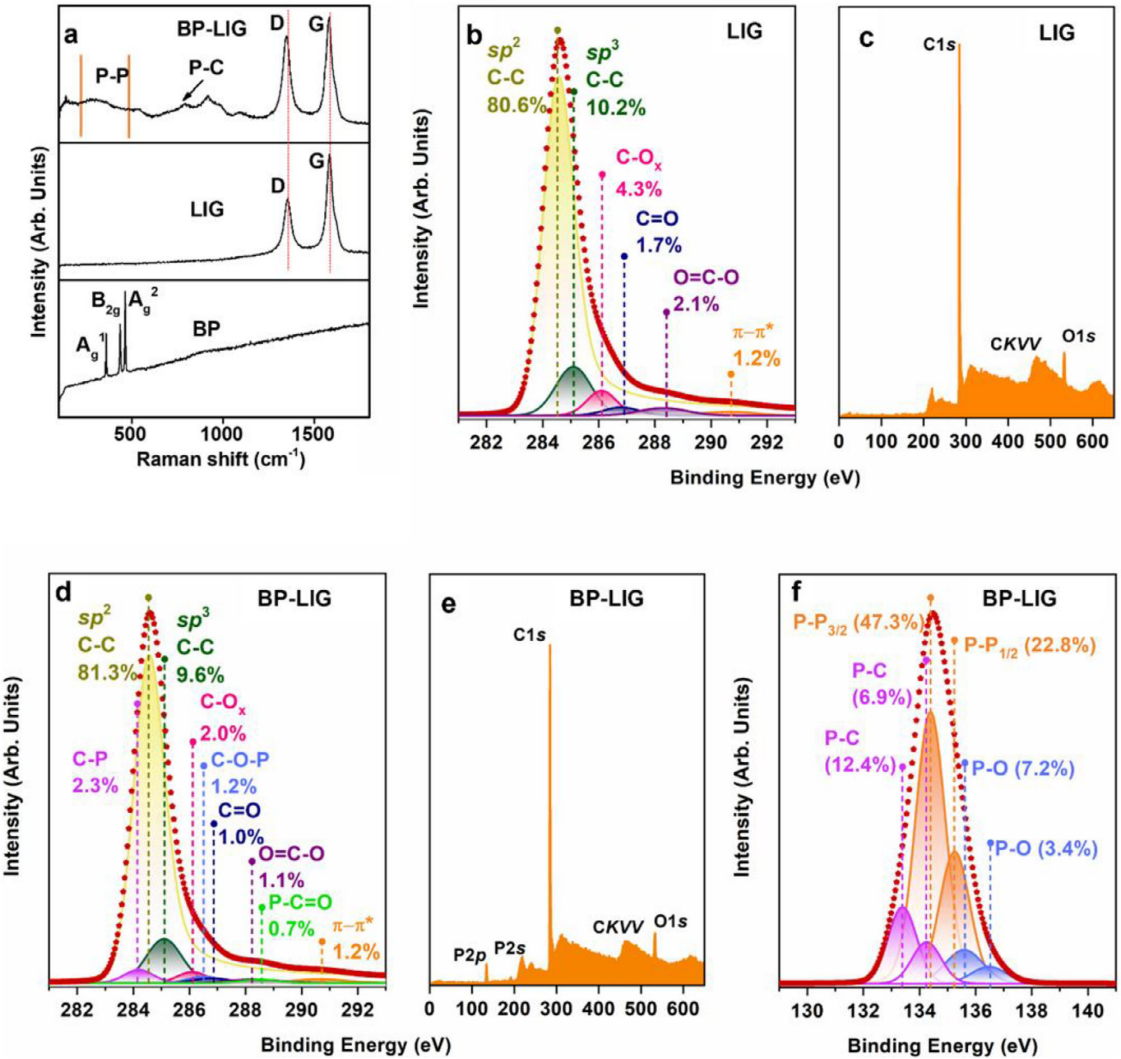
Physical characterizations. a) Raman spectra of BP, LIG, and BP‐LIG hybrid. High‐resolution X‐ray photoelectron spectra: **b)** C1s, **c)** survey spectra for LIG, **d)** C1s, **e)** survey spectra **f)** P2p, for BP‐LIG.

**Figure** **3**a compares the Raman spectra of the bulk BP, LIG, and BP‐LIG hybrid. The BP exhibits three Raman active modes centered around ≈464, ≈436, and ≈360 cm^−1^, which are assigned to A_g_^2^, B_2g,_ and A_g_^1,^ respectively.^[^ ^40^, ^41^ ^]^ In the Raman spectrum of pristine LIG, two intense bands are observed at ≈1355 and 1582.5 cm^−1^, corresponding to the D and G modes, respectively.^[^ ^38^ ^]^ The BP‐LIG hybrid retains the signature of Raman active modes of both BP and LIG. The Raman spectrum of BP‐LIG shows a broad peak in the region 250 to 550 cm^−1^, indicative of P─P bonds.^[^ ^23^ ^]^ Interesting to note that the P─P band intensity is significantly decreased for BP‐LIG as compared to BP due to P─P bond breakage and formation of new P─C bonds. The broad band envelope in the range of 650 to 800 cm^−1^ belongs to the P─C bond stretching modes.^[^ ^42^, ^43^ ^]^ The peaks in the 1200 to 1800 cm^‐−1^ region are the characteristics of defective graphitic carbon. `The D mode is centered around 1347 cm^−1,^ indicating the breathing mode of aromatic carbon, while the G band around 1580 cm^−1^ is indicative of E_2g_ symmetrical bond stretching of sp^2^ carbon atoms.^[^ ^42^, ^43^ ^]^ The downshifting of the G band of BP‐LIG hybrid to a lower wavenumber compared to LIG can be explained based on π–p* conjugation, also suggesting the formation of P─C bonds.^[^ ^23^ ^]^ The P─C bonds play a highly important role in dictating the electrochemical performance of LIB.^[^ ^23^ ^]^ The findings from Raman are further verified by the synchrotron‐based XPS and NEXAFS study discussed next.

Figure 3b–f represents the high‐resolution C1s (Figure 3b,d), P2p (Figure 3f), and survey (Figure 3c,e) photoelectron spectra of the LIG and BP‐LIG. The presented photoelectron spectra were acquired with an excitation energy of 730 eV. The XPS survey spectrum of LIG confirms the results on the chemical composition of this sample obtained by EDX spectroscopy (as shown in Figure 2). The C1*s* photoelectron spectrum of LIG has a complex structure. It is well described by a deconvolution with six components. Plotted in dark yellow, the main component with a binding energy (BE) of 284.5 eV (*BE *= 284.5 eV) has an asymmetrical shape and is described using the Doniach–Sunjic function.^[^ ^44^ ^]^ Hence, it can be identified with the C═C phase with *sp* ^2^ hybridization of the valence electron states of the carbon atoms, typical for graphene and HOPG.^[^ ^45^ ^]^ The contribution of this component to the C1s photoelectron spectrum of LIG is dominant since the relative intensity of this component is equal to ≈81%. This fact, together with the presence in the spectrum of the π–π* satellite with BE = 290.6 eV (plotted in orange), indicates a high quality of the fabricated LIG.^[^ ^46^ ^]^ The low relative intensity (10.2%) of the component with BE = 285.1 eV (plotted in green), which is identified with *sp* ^3^ carbon atoms, also indicates a small number of defects in LIG. In contrast to the main component, this component, along with the three other higher‐energy components, has a symmetrical shape. These three components with BE = 286.1 eV (plotted in pink), BE = 286.8 eV (plotted in navy), and BE = 288.3 eV (plotted in purple) arise due to chemical bonding between carbon and oxygen atoms. This interaction leads to the formation of oxygen‐containing functional groups and is accompanied by charge transfer from carbon atoms to oxygen atoms since oxygen atoms have greater electronegativity compared to carbon atoms. These components can be identified with C─O_x_, C═O (carbonyl), and O═C─O (carboxyl).^[^ ^47^ ^]^ Their relative content in LIG is 4.3%, 1.7%, and 2.1%, respectively. Therefore, it is logical to conclude that the main share of oxygen in LIG is a result of surface contaminations.

As in the EDX spectrum of BP‐LIG (Figure 2), signals from phosphorus, carbon, and oxygen atoms can be easily seen in the survey X‐ray photoelectron spectrum of BP‐LIG (Figure 3e). The C1c photoelectron spectrum of BP‐LIG is a complex structure with its current components (Figure 3d). The main component (plotted in dark yellow) is located at BE  =  284.5 eV and has an asymmetrical shape described using the Doniach–Sunjic function, just like in the case of LIG. Thus, it is identified with C═C *sp* ^2^ hybridized electron states. Its relative intensity is only slightly greater than in the case of LIG, namely 81.3%. The relative intensity of the π–π* satellite with BE  =  290.6 eV (plotted in orange) has the same value as in LIG. At the same time, the relative intensity of this component with BE  =  285.1 eV (plotted in dark green), corresponding to C─C *sp* ^3^ hybridized electron states, has a slightly lower value than in LIG, namely 9.6%. Thus, it is obvious that BP‐LIG retains the structure of LIG with the predominant type of C═C *sp* ^2^ hybridization, which is characteristic of the graphene mesh. In addition, six more components with symmetrical shapes are clearly distinguishable in the C1s spectrum of BP‐LIG. As in the case of LIG, three of them with BE  =  286.1 eV, BE  =  286.8 eV, and *BE* = 288.3 eV can be identified with oxygen‐containing functional groups, namely C─O_x_ (plotted in pink), C═O (plotted in navy), and O═C─O (plotted in purple). It is important to emphasize that the relative intensities of these components in BP‐LIG are almost half (2.0%, 1.0%, and 1.1%, respectively) compared to LIG. In addition, a significant difference between the С1s photoelectron spectrum of BP‐LIG and the С1s photoelectron spectrum of LIG is the appearance of three additional components. One of them, in contrast to all other symmetric components, is located on the side of lower binding energies from the main component and has the binding energy of *BE* = 284.1 eV. This component is a result of the formation of phosphorus‐containing functional groups, namely C–P groups.^[^ ^48^, ^49^ ^]^ By analogy with intercalated graphene and intercalated single‐walled carbon nanotubes, such formation of C–P groups is accompanied by charge transfer from phosphorus atoms to carbon atoms due to their lower electronegativity. ^[^ ^50^ ^]^ The relative intensity of this component is equal to ≈2.3%. The other two components are located on the side of high binding energies from the main component and have the binding energies of BE  =  286.4 eV and BE  =  288.7 eV. The relative intensities of these components are equal to ≈1.2% and ≈0.7%, respectively. These components are also a consequence of the formation of phosphorus‐containing functional groups, namely C─O─P and P─C═O groups, respectively.^[^ ^48^ ^]^ This confirms the conclusion made above from the analysis of the SEM micrograph (Figure 2b) that BP particles are bound to graphene. It is very important to emphasize that such bonding takes place without corrugation and deformation of the LIG graphene mesh, as evidenced by the values of the relative concentrations of *sp* ^2^ and *sp* ^3^ components in the C1s photoelectron spectrum of BP‐LIG compared to the C1*s* photoelectron spectrum of LIG. The appearance of C─O─P and P─C═O groups in the BP‐LIG structure is a consequence of the replacement of some of the oxygen atoms on the BP‐LIG surface with phosphorus atoms, thereby slightly improving the structure of the graphene network compared to LIG. Hence, it can be assumed that BP particles are encapsulated between LIG layers, and are not embedded in the graphene mesh, replacing carbon atoms there. Moreover, covalent bonds are formed between carbon, oxygen, and phosphorus atoms in the case of C─O─P and P─C═O groups.

The conclusions drawn from the analysis of the C1*s* photoelectron spectrum of BP‐LIG are also confirmed by the structure of the P2*p* photoelectron spectrum (Figure 3e). The P2*p* photoelectron spectrum of BP‐LIG has a complex structure, as does the C1*s* photoelectron spectrum of BP‐LIG. It is well described by a deconvolution with six components. These six components are parts of three doublets. The existence of these three doublets is a consequence of the spin‐orbit splitting of the P 2*p* states. Therefore, each doublet has two components, namely P2*p*_1/2_ and P2*p*_3/2_. The splitting interval between P2*p*_1/2_ and P2*p*_3/2_ components is equal to ≈0.86 eV, and the areas under these peaks are related as 1 to 2. The two most intense components, with BE = 134.4 eV and BE = 135.3 eV (plotted in light orange), can be identified with the P2*p*_3/2_ and P2*p*_1/2_ electron states of elemental phosphorous. The relative intensity of this doublet is equal to ≈70.1%. The other two components, with BE = 133.4 eV and BE = 134.3 eV (plotted in light purple), can be identified with the P2*p*_3/2_ and P2*p*_1/2_ electron states of the P–C phase.^[^ ^51^ ^]^ The relative intensity of this doublet is equal to ≈19.3%. Two more components with BE = 135.6 eV and BE = 136.5 eV (plotted in light blue) can be identified with the P2*p*_3/2_ and P2*p*_1/2_ electron states of the P‐O phase.^[^ ^48^ ^]^ The relative intensity of this doublet is equal to ≈10.6%. The excitation energy of 730 eV, which was used in the analysis of LIG and BP‐LIG, corresponds to a probing depth of ≈1.9 nm for P 2*p* states. If we take into account that the sizes of BP particles have diameters of ≈100 nm, then as a result of the analysis, we are able to identify the contribution of both the bulk component of BP particles and the functional groups on their surfaces.

**Figure 4:**
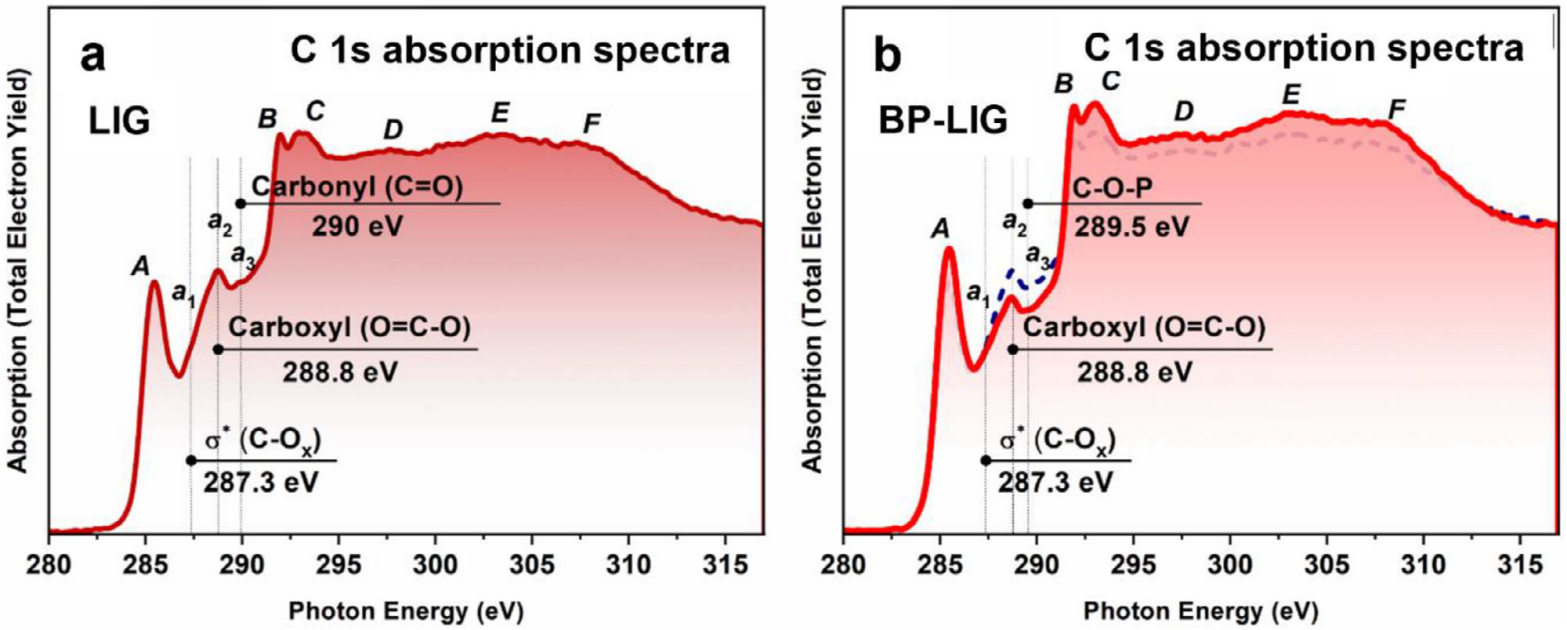
NEXAFS spectroscopy results. C1*s* X‐ray absorption spectra of **a)** LIG and **b)** BP‐LIG and LIG (plotted in navy dash).

The results obtained from the characterization of both LIG and BP‐LIG samples by X‐ray photoelectron spectroscopy were also confirmed by NEXAFS spectroscopy. **Figure** **4**a shows the C1s X‐ray absorption spectrum of the LIG sample, which perfectly reproduces all the main features of the HOPG and graphene spectra reported earlier.^[^ ^52^ ^]^ First of all, this concerns the absorption bands *A* and *B‐C*, which are associated with transitions of C1*s* electrons to free states of π‐ and σ‐symmetry of the conduction band, formed from π2*р*_z_‐ and σ2*p*_x,y_‐states of carbon atoms.^[^ ^53^ ^]^ As well in the LIG spectrum, absorption bands *D*–*F* are observed, which reflect electronic transitions to free σ‐states of the conduction band.^[^ ^54^ ^]^ It is important to emphasize that in the case of LIG, similar differences are observed between the positions of peaks A‐B (ΔЕ_А–В_ = 6.5 eV) and A–C (ΔЕ_А–С_ = 7.6 eV) and the full width at half maximum of peak A (FWHM = 1.7 eV) as in the case of graphene (ΔЕ_А–В_ = 6.4 eV, ΔЕ_А–С_ = 7.4 eV, FWHM = 1.55 eV) and HOPG (ΔЕ_А–В_ = 6.4 eV, ΔЕ_А–С_ = 7.4 eV, FWHM = 1.15 eV). In addition to this, the LIG spectrum lacks a step before resonance A with hv = 283.7 eV,^[^ ^50^ ^]^ which indicates a few‐layer graphene structure of the LIG,^[^ ^44^ ^]^ as demonstrated by the SEM (Figure 2). All these findings indicate a high quality of the obtained LIG, with predominant *sp* ^2^ hybridization of graphene mesh. At the same time, the broadening of band A is a consequence of a slight curvature of flat graphene layers and a decrease in their symmetry in LIG, the presence of a small number of defects in graphene sheets,^[^ ^44^ ^]^ as well as the chemical bonding of carbon atoms with oxygen atoms. The presence of such chemical bonding manifests in the LIG spectrum in the form of three additional features a_1_ (hv = 287.3 eV), a_2_ (hv = 288.8 eV), and a_3_ (hv = 290 eV), between the π‐ and σ‐resonances. They are caused by transitions of C1s electrons to free C2p states on the oxidized areas of the LIG surface with the formation of C─O_x_, carboxyl (O═C─O) and carbonyl (C═O) bonds,^[^ ^55^ ^]^ respectively. As shown above, the XPS spectra (Figure 3a,b) confirm the existence of a small amount (≈8%) of chemically bound oxygen in LIG also.

Figure 4b presents the C1s X‐ray absorption spectrum of the BP‐LIG sample (plotted in red). Herein, the C1s X‐ray absorption spectrum of the LIG sample is plotted in dashed navy. The spectrum of BP‐LIG is similar to the spectrum of LIG and also perfectly reproduces all the main features of the spectra of HOPG and graphene, namely the absorption bands A‐F.^[^ ^56^ ^]^ Simultaneously, this spectrum is characterized by a slightly higher intensity of the band *A* compared to the LIG spectrum, namely I(A_BP‐LIG_)/I(A_LIG_) = 1.11. In addition to this difference, the FWHM of peak A, along with the difference between the positions of peaks A and C, are slightly smaller than those in the case of LIG, namely FWHM = 1.6 eV and ΔE_A–C_ = 7.5 eV. These three facts indicate the higher quality of graphene mesh in case of BP‐LIG compared to LIG. The spectrum of BP‐LIG also exhibits three low‐intensity features a_1_ (hv = 287.3 eV), a_2_ (hv = 288.8 eV) and a_3_ (hv = 289.5 eV) between the π‐ and σ‐resonances, which are also due to transitions of C1s electrons to free C2p‐states on the oxidized surface areas of BP‐LIG with the formation of (C─Ox), carboxyl (O═C─O), and carbonyl (C═O) bonds, respectively.^[^ ^55^ ^]^ It is important to note that the intensity of these features is much less in the spectrum of BP‐LIG than in the case of LIG one. This observation is in good agreement with the XPS spectra of BP‐LIG (Figure 3d–e), which exhibit a lower amount (≈5.3%) of chemically bound oxygen in BP‐LIG. Nevertheless, it is especially important to emphasize that the feature a_3_ (hv = 289.5 eV) in the BP‐LIG spectrum is shifted by 0.5 eV toward lower photon energies. In our opinion, this observation may be a manifestation of the formation of C─O─P bonds, which were revealed during the analysis of the C1s X‐ray photoelectron spectrum of BP‐LIG (Figure 3d). However, we were unable to detect in the C1s X‐ray absorption spectrum of the BP‐LIG sample a feature with *hv* = 288.4 eV, which was previously interpreted as a covalent bond between carbon and phosphorus atoms.^[^ ^57^ ^]^ This observation is consistent with our findings from the analysis of the C1s X‐ray photoelectron spectrum of BP‐LIG (Figure 3d) that the component corresponding to the formation of C─P bonds has a lower binding energy compared to the component associated with the formation of C═C *sp* ^2^ bonds, indicating a non‐covalent type of bonding between carbon and phosphorus atoms. Thereby, our results obtained by core‐level spectroscopy are in very good agreement with the conclusions made above in our work when analyzing the morphology of the BP‐LIG sample using SEM and EDX methods, namely, that the BP particles are located between the graphene sheets, forming heterostructures with the formation of covalent P─O─C bonds between the phosphorus, oxygen and carbon atoms, which prevents the self‐restacking of both 2D BP and graphene sheets. Besides this, there is also a weak (π–π) interaction between BP particles and graphene sheets, which also allows them to be ordered. As a result, such heterostructures are more compact and more ordered compared to LIG. This is also consistent with some of the conclusions made in the publication.^[^ ^48^ ^]^

### 2.2 Electrochemical Part

**Figure 5:**
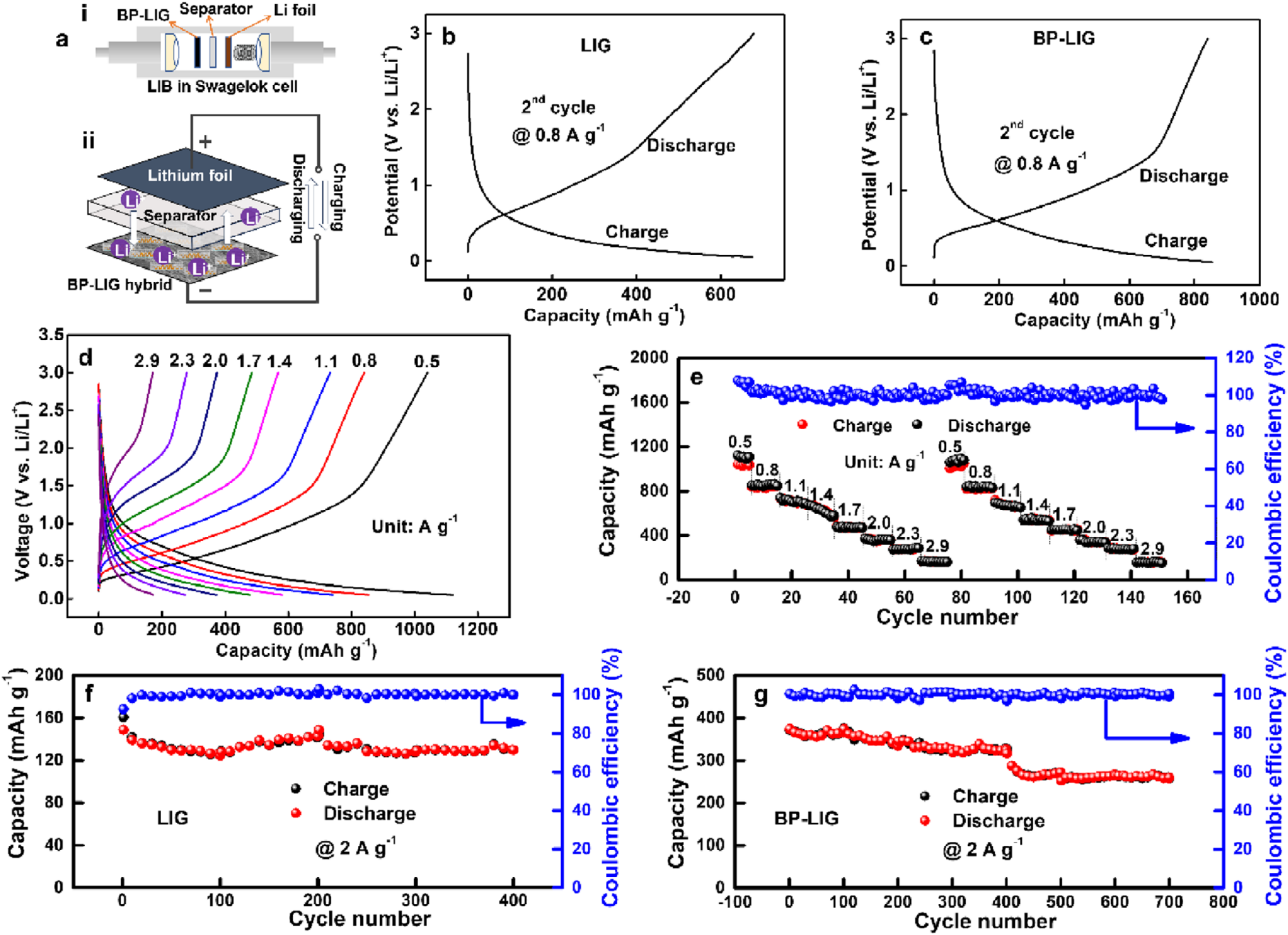
Electrochemical performance of BP‐LIG hybrid Li‐ion cells. a) Schematic diagram of Li‐ion cell, which consists of a BP‐LIG anode, Li cathode, and Li‐based electrolyte. b,c) Voltage profile of LIG and BP‐LIG hybrid at a current density of 0.8 A g^−1^. d) Voltage profile of a BP‐LIG hybrid at different current densities. e) Rate performance of the BP‐LIG hybrid at different current densities. f,g) Cycling stability and Coulombic Efficiency over 400 cycles for LIG and BP‐LIG hybrid, respectively.

The BP‐LIG electrodes were assembled against a lithium foil and glass fiber separator using a two‐electrode Swagelok cell (**Figure** **5**a‐i,ii) and cycled between potential windows of 0.05–3 V. Figure 5b,c, represent the typical discharge/charge voltage profile characteristics of LIG and BP‐LIG electrodes at a current density of 0.8 A g^−1^. The voltage profiles confirm the intercalation of Li^+^ ions into the electrode layers, a behavior similar to that observed in photothermally reduced graphene structures.^[^ ^58^ ^]^ Here, all the fabricated half‐cells were discharged and charged at 0.8 A g^−1^ to form a smooth solid electrolyte interphase surface on the LIG and BP‐LIG electrode. Then, we record the discharge and charge profile with the designated rate from the second cycle onward.^[^ ^59^ ^]^ The voltage profile of Figure 5b,c indicates that BP‐LIG has improved reversibility as compared to LIG in the lithiation and delithiation process of Li^+^ ions once the solid electrolyte interphase is formed. The LIG and BP‐LIG electrodes delivered a reversible charge capacity of 674 and 860 mAh g^−1^ at 2nd cycle, respectively. The nano BP particles encapsulated in 3D porous LIG matrix can assist charge transfer due to the shortened charge transfer pathways observed similarly in the BP‐carbon nanotube hybrid.^[^ ^60^ ^]^ The capacity improvement can also be attributed to increased *d* spacing of graphene due to nano BP insertion^[^ ^61^ ^]^ and Li^+^ ion intercalation on both sides of graphene layers corresponding to the formation of Li_2_C_6_.^[^ ^62^, ^63^ ^]^

The capacity retention trend after 10 cycles was also monitored at 0.8 A g^−1^ (Figure S4, Supporting Information). LIG loses 14% of discharge capacity even after 10 discharge/charge cycles, whereas for BP‐LIG, the capacity remains constant till the 10th cycle. To understand the extent of improvement of BP‐LIG, electrode, the rate capability tests of BP‐LIG at various current densities were carried out. All the BP‐LIG half cells were cycled at a current density from 0.5–2.9 A g^−1^ and then repeated the cycles again. The completed rate characteristics are shown in Figure 5e where the half cells were run for 10 cycles at each current density. The specific capacity of the BP‐LIG electrode was calculated as 1122 mAh g^−1^ at 0.5 A g^−1^. When the current rate returned to 0.5 A g^−1^ after 75 cycles, the specific capacity was recovered 94.5% (1060 mAh g^−1^), showing excellent rate capability.

To demonstrate the advantage of BP‐LIG hybrid over LIG, the cyclic performance of BP‐LIG and LIG was studied with similar experimental parameters for over 500 discharge/charge cycles. The specific capacity of the BP‐LIG electrode remains at 326 mAh g^−1^, after 400 cycles (Figure 5g), which is much higher than that of LIG 130 mAh g^−1^ (Figure 5f). After 700 cycles the specific capacity of the BP‐LIG electrode falls to 259 mAh g^−1^. Note that Coulombic efficiencies of the BP‐LIG hybrids were recorded ≈100% over 700 cycles. Such exceptional stability of the BP‐LIG anode is quite striking and has not been demonstrated in BP‐based hybrids.^[^ ^21^, ^60^, ^64^ ^]^ The stability performance of the reported phosphorus‐carbon composite structure is now compared with our results (**Table** **1**).

**Table 1:** LIB performance comparison of BP‐LIG anode with other reported literature.

BP composite with carbon	Initial discharge capacity	Cyclic stability	Refs.
Sandwiched thin‐film anode of chemically bonded black phosphorus/graphene	1633 mAh g^−1^ @ 0.1 A g^−1^ after 10th cycle	1401 mAh g^−1^ @ 0.1 A g^−1^; after 200 cycles	[65]
BP and graphite	2786 mAh g^−1^ @ 0.2C	1849 mAh g^−1^ @ 0.2C; after 100 cycles	[23]
Black Phosphorus (BP)‐ Titanium dioxide (TiO_2_)‐ Carbon (C) nanocomposite	1581.1 mAh g^−1^ @ 0.1 A g^−1^; 1st cycle	935.8 mAh g^−1^ @ 2 A g^−1^; after 300 cycles	[66]
BP on the conductive carbon paper (BP‐CP)	2168.8 mAh g^−1^ @ 0.1C; 1st cycle	1677.3 mAh g^−1^ @ 0.1C; after 200 cycles	[67]
BP‐CNT composites	2073 mAh g^−1^ @ 0.1C; 1st cycle	1681 mAh g^−1^ @ 0.2C; after 400 cycles	[68]
3D red phosphorus/sheared CNT sponge	1600 mAh g^−1^ @ 0.1 A g^−1^	1398.5 mAh g^−1^ @ 0.1 A g^−1^; after 200 cycles	[69]
Crumpled Nitrogen‐Doped Graphene‐Wrapped Phosphorus Composite	1340 mAh g^−1^ @ 3.9 A g^−1^	1470 mAh g^−1^ @ 1.3 A g^−1^; after 300 cycles	[70]
Red phosphorus‐carbon (P/C) composite nanostructure	1684 mAh g^−1^ @ 3 mA cm^−2^	1625 mAh g^−1^ @ 0.86 mA cm^−2^; after 500 cycles	[71]
Red phosphorus anchored on carbon nanotubes	886 mAh g^−1^ @ 2 A g^−1^	960 mAh g^−1^ @ 0.2 A g^−1^; after 120 cycles	[72]
Black phosphorus@CNTs Hybrid	2229 mAh g^−1^ @ 0.1 A g^−1^	1088 mAh g^−1^ @ 0.1 A g^−1^, after 100 cycles	[60]
BP and graphene oxide, with the output coated *in situ* by polyaniline (BP‐GO‐PANI)	2380 mAh g^−1^ @ 0.1 A g^−1^	652 mAh g^−1^ @ 0.1 A g^−1^, after 50 cycles	[73]
Phosphorene–graphene composite	2763 mAh g^−1^ @ 0.1 A g^−1^	725 mAh g^−1^ @ 0.5 A g^−1^, after 200 cycles	[74]
BP‐ highly conductive graphene sheets	≈940 mAh g^−1^ @ 0.1 A g^−1^	402 mAh g^−1^ @ 0.5 A g^−1^, after 500 cycles	[19]
BP‐LIG	1122 mAh g^−1^ @ 0.5 A g^−1^	326 mAh g^−1^ @ 2 A g^−1^, after 400 cycles	This work

To verify the reproducibility, we have prepared three set electrodes for each sample type (LIG and BP‐LIG) and carried out the cyclic stability test upto 100 cycles at 2 A g^−1^. For the pure LIG samples, the initial cycles exhibit relatively large deviations, with error bars reaching up to 24% (Figure S4 and S5, Supporting Information). However, the variability decreases over time and stabilizes below 10% in the later cycles. In contrast, the BP‐LIG electrodes show much smaller fluctuations from the beginning, with an average deviation of ≈7% for both the charging and discharging processes throughout the 100 cycles (Figure S5, Supporting Information). These results indicate improved cycling stability and better reproducibility for the BP‐LIG system compared to pure LIG.

The heterogeneous architecture prevents graphene restacking, maintaining accessible lithium storage sites throughout cycling. Graphene layers function as elastic buffers, accommodating black phosphorus's substantial volume changes (≈300%) during lithiation/delithiation processes.^[^ ^23^ ^]^ The 2D layered structure facilitates rapid lithium‐ion diffusion while minimizing transport distances across the electrode matrix. Graphene passivation mitigates black phosphorus's environmental degradation, addressing a fundamental limitation of phosphorene‐based materials. To reveal the cause of good stability over the long cycles, *ex situ* SEM was carried out to study the structural evolution of the anode during various cyclic periods.

**Figure 6:**
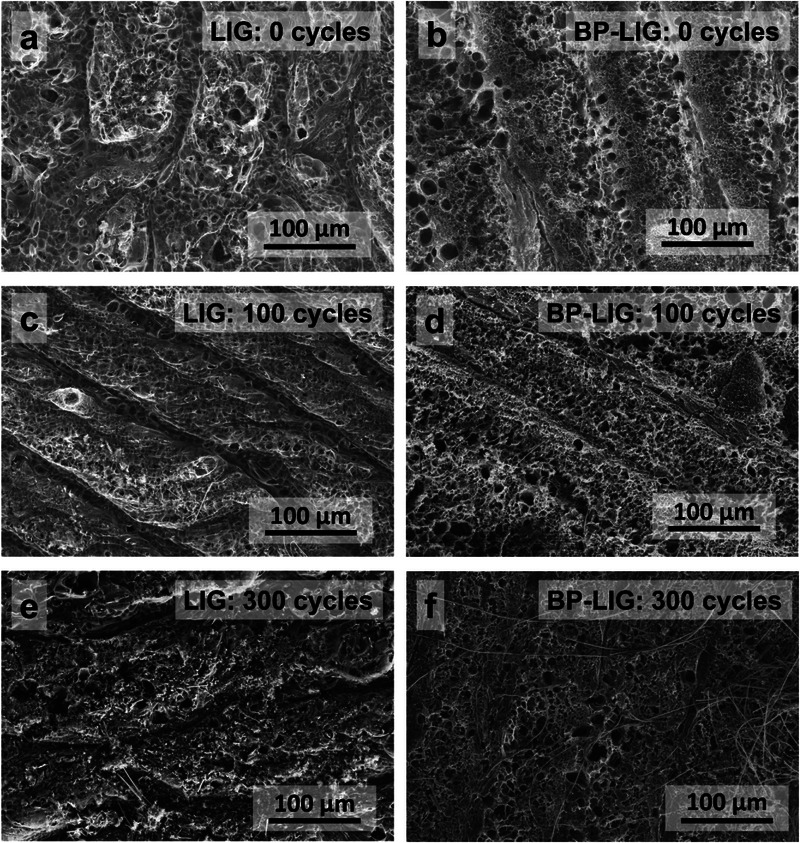
Ex situ SEM images of a,c,e) LIG and b,d,f) BP‐LIG anode captured after the n^th^ (0, 100, and 300) cycle.

**Figure** **6**a–f illustrates the morphological evolution of LIG and BP‐LIG hetrostructure at different stages of the long cyclic process. The LIG electrode transformed from a smooth porous structure to a rougher surface after 300 cycles, with visible fragmentation and debris scattered across the electrode surface. Whereas, during the continuous lithiation/delithiation process, the BP‐LIG hybrid maintains an intact and compact structure, and there is no obvious fragmentation on the electrode surface before and after the cycles. Note that, bulk BP materials with larger 2D sheets typically form Li_3_P phase during the lithiation process, resulting in a significant volume expansion of 300%. The expansion is reversed during delithiation cycles. The repeated expansion/shrinkage during prolonged lithiation/delithiation cycles causes the original BP structure to collapse.^[^ ^60^ ^]^ This mechanical stress leads to the fracture and disrupts the continuous contact between the active material and the current collector, ultimately degrading performance. In our case, the strong covalent bonding (P─C, P─O─C) between nanoscale BP particles and LIG network enhances the structural stability, enabling sustained performance over extended cycles.

### 2.3 Theoretical Insight of the BP‐LIG Heterostructure and Electrochemical Processes

Multiscale modelling has been demonstrated as an effective simulation tool for the description of electrochemical processes occurring in battery systems.^[^ ^75^ ^]^ Here, the phosphorene‐graphite heterostructures were studied using *ab initio* DFT modelling and REAX FF molecular dynamics.

**Figure 7:**
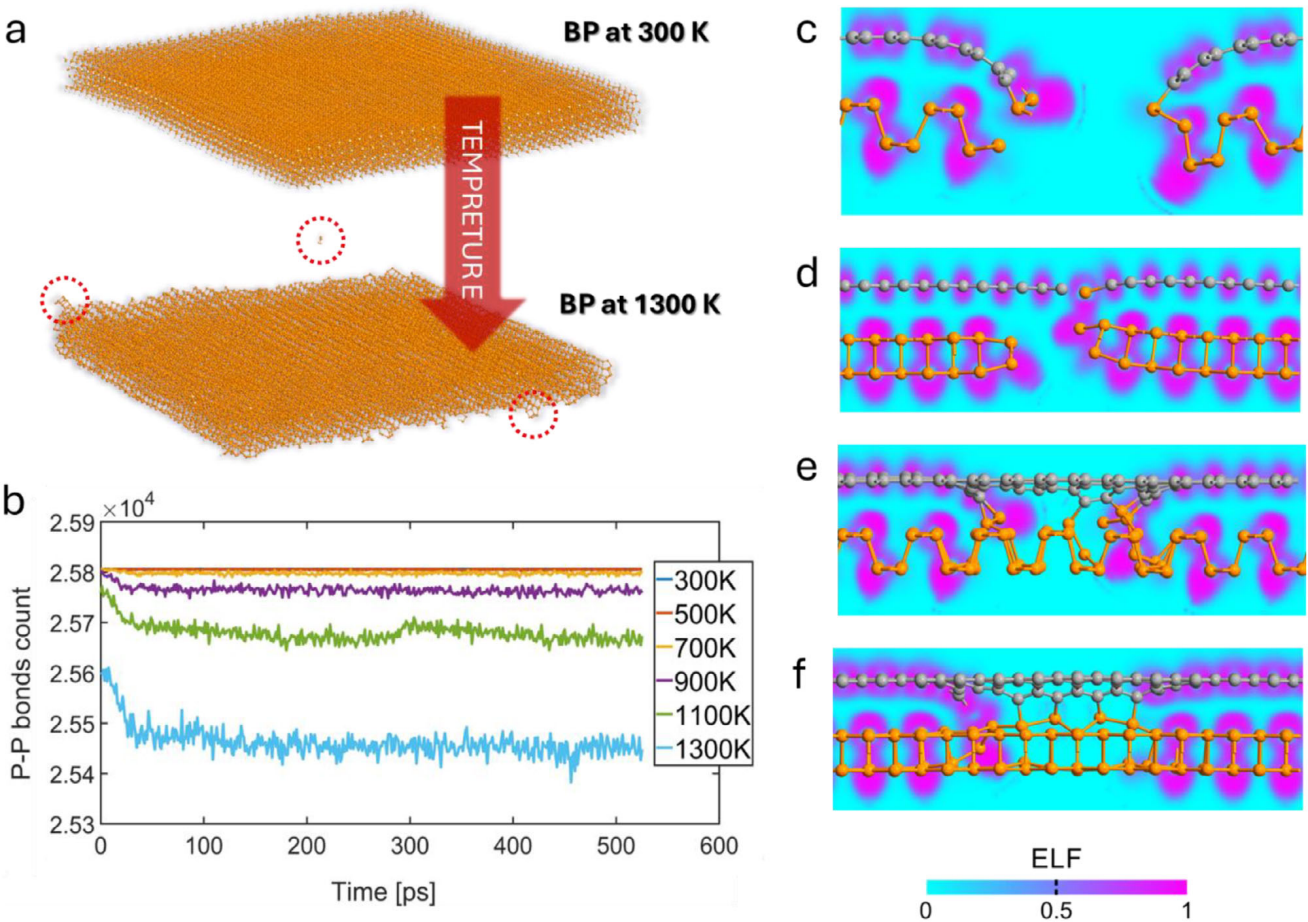
Thermal stability simulation. a) The atomic model of the 15 nm nanoflake of three layer phosphorene in 300 and 1300K b) time evolution of P─P bond number in 15 nm phosphorene flake versus temperature c) the optimal geometry of phosphorene and graphene covalent bonding through phosphorene zigzag edge with ELF distribution d) the optimal geometry of phosphorene and graphene covalent bonding through phosphorene armchair edge with ELF distribution e) the surface reconstruction around hole in graphene‐phosphorene “sandwich” configuration, with ELF distribution along armchair direction and f) zigzag direction.

An atomic model of the phosphorene nanostructure was constructed to provide insight into the process of covalent bonding between layers of the material. The model utilised for thermal stability simulation represents the 15 nm flake, which consists of three layers of T_b_ stacked^[^ ^76^ ^]^ phosphorene with hydrogen‐terminated edges (**Figure** **7**a). The structure was thermalised in 300 K for 500 ps with canonical ensemble (NVT) molecular dynamics in order to create a proper starting configuration. The following protocol was implemented to investigate its stability: The NVT molecular dynamics simulation was conducted in 500 ps intervals after each assembly temperature was raised by 200 K during a 20 ps interval. The procedure was continued until the assembly reached a temperature of 1300 K, resulting in a 3 ns molecular dynamics trajectory. According to the literature, such temperatures are typical for material inside the laser spot during the laser ablation process.^[^ ^36^, ^77^, ^78^ ^]^ The interatomic forces were described using REAX FF force field designed for phosphorene nanostructures.^[^ ^79^ ^]^ The simulation was conducted within the LAMMPS package framework.^[^ ^80^ ^]^

It was noted that the structure undergoes edge degradation, which begins after the temperature exceeds 700 K. This process starts with the release of the hydrogen adsorbed at the edges; however, it also leads to breaking P‐P bonds and the creation of multiple free radical sites. This process is presented in Figure 7a. The red circles indicate the radical sites and free phosphorus atoms released from the edges in the thermal decomposition process. In order to quantify this process, the time evolution of the number of P‐P bonds was presented in Figure 7b. It indicates that thermal degradation begins at temperatures above 700 K and is significantly accelerated when the temperature exceeds 1100 K. This finding is consistent with observations presented in.^[^ ^81^ ^]^

To examine the configuration of the edge reconstruction, the DFT geometry optimisation was conducted. This approach was required to achieve a correct description of the interlayer covalent bonding process since the currently available phosphorene REAX force field does not properly describe bonds other than P─P and H─P.^[^ ^82^ ^]^

In this part of the simulations, the three atomic models were used. First, representing the interaction of zigzag edges (Figure 7c), was constructed by interfacing 3x6 single‐layer phosphorene with 4 × 6 single‐layer graphene (dimension in lattice constants). The atoms from the central part of the cell were removed, creating a gap with one lattice constant width. The second model represents armchair edges interaction (Figure 7d) and was constructed similarly by interfacing a 9 × 3 phosphorene layer with a 12 × 3 graphene. The third model (Figure 7e) represents a finite hole defect and was constructed by interfacing 9 × 7 phosphorene with 12 × 7 graphene. The size of the hole was 3 × 2. In each model, the starting configuration of the phosphorene edge was defective according to molecular dynamics results.

The models of the structure for DFT calculations were chosen to represent the interaction between the edges of phosphorene and graphene layers. The common approach for designing the 2D heterostructure atomic models is molecular cluster approximation, as shown in;^[^ ^83^ ^]^ however, the mentioned work focuses on the study of the lithium‐ion diffusion over the defect‐free surface. Because of the small radius of Li ion, the results of adsorption energy and diffusion calculations are not affected by the finite and relatively small sizes of flakes used in calculation using molecular cluster approximation, leading to the same results as calculations using periodic boundary conditions.^[^ ^83^, ^84^, ^85^ ^]^ In our simulations, the interaction between edges of the structures was the primary concern, and these processes are dependent on the size of the nanoflake.^[^ ^86^ ^]^ Because the direct DFT simulation of the flakes with the sizes of 280 nm (size estimated by our DLS measurements) was impossible due to extraordinary computational complexity, the models were designed using periodic boundary conditions. The size of the supercell used was chosen to mitigate the effect of periodic image interactions between atoms.

The geometry of all three models was optimized using the L‐BFGS algorithm to minimize their potential energy, which triggers the edge reconstruction within the model. The final geometries of the models after the optimisation were shown in Figure 7c,f, suggesting that the edges will be connected by the P─C covalent bond.

The electron localisation function (ELF) distribution was calculated to prove that the interaction between edge atoms from phosphorene and graphene is covalent. The results presented in Figure 7c,d suggest that covalent bonding exists between layers in both configurations. This was not evident from the bond length analysis, since the distance between P and C atoms was 2.37 Å. The results achieved for the small hole defect (Figure 7e,f) were consistent with this observation.

**Figure 8:**
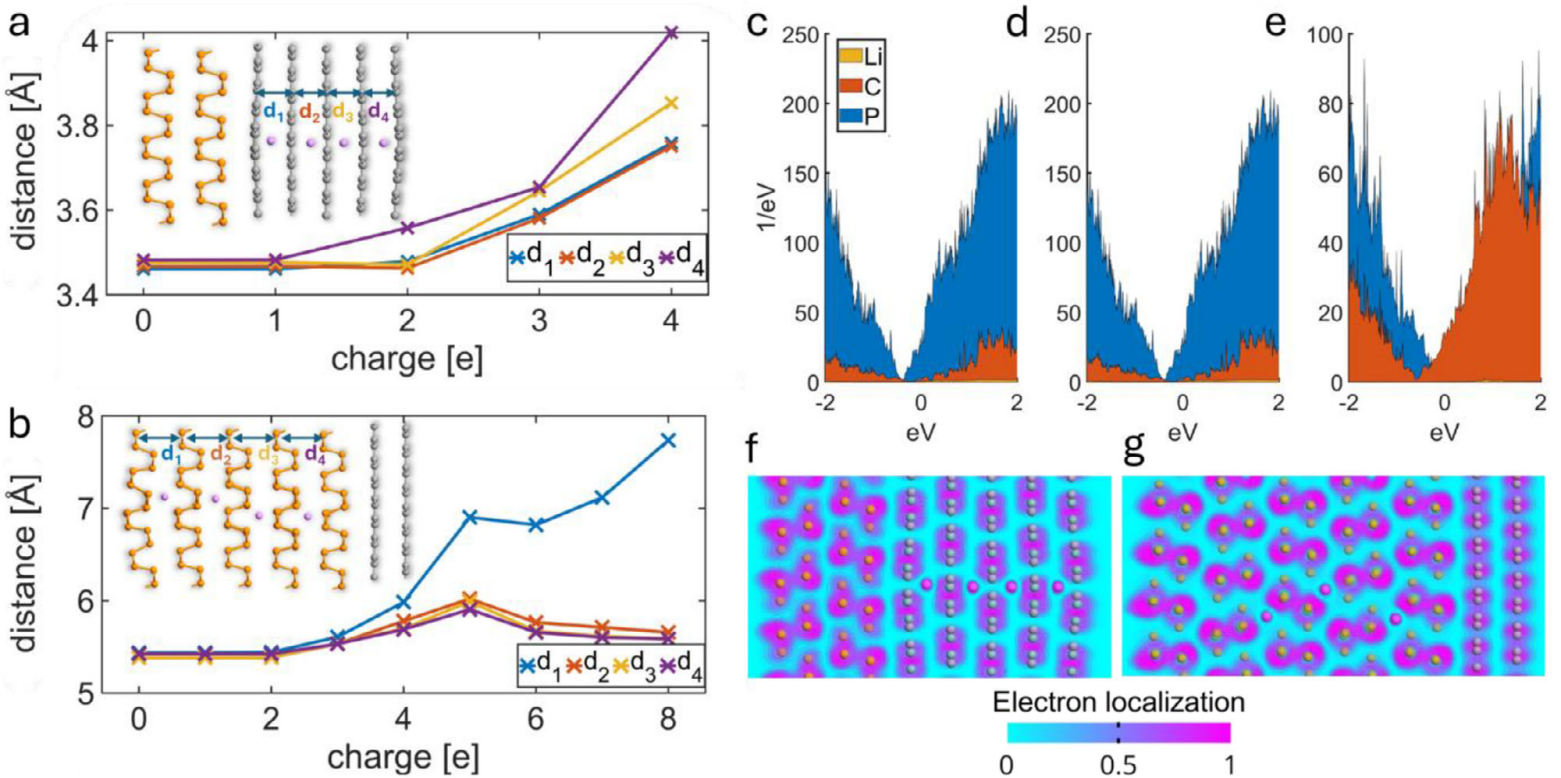
Charged structures simulation. a) The effect of charge on the interlayer distance after intercalation in graphene b) and phosphorene c) the projected density of states for structure with 5 phosphorene and 2 graphene layers: 4 d) and 8 e) Li^+^ ions, and for structure with 2 phosphorene and 5 graphene layers with 4 Li^+^ ions f) the electron localization function distribution for structure with 2 phosphorene and 5 graphene g) the electron localization function distribution for structure with 2 phosphorene and 5 graphene.

The effect of structure intercalation by Li^+^ ions was also examined. For this purpose, the atomic models of LIB were constructed. They were created by interfacing 3 × 4 phosphorene supercell with 4 × 4 graphene supercell. Two different ratios of phosphorene to graphene number of layers were tested: 2 to 5 (**Figure** **8**f) and 5 to 2 (Figure 8g).

The geometry of the models was optimised using a similar DFT approach as in the models of edge interaction. The projected density of states (PDOS) was calculated for all models. Figure 8c,e presents the PDOS for the structure with a 5 to 2 phosphorene/graphene layer ratio with intercalation levels equal to 4 Li^+^ and 8 Li^+^ in the phosphorene structure. Figure 8f presents the PDOS for the structure with 2 to 5 phosphorene/graphene layer ratio and 4 Li^+^ ions in the graphene structure. All structures represent metallic properties thanks to interfacing with graphene, which is desired for battery applications.^[^ ^75^ ^]^

The ELF was analyzed for both examined structures. The ELF is close to 1 between the atoms forming the heterostructure layers, which indicates covalent bonding. Electron localization is close to zero around each Li atom, which proves that no bond formation occurs, and the model describes the proper intercalation process.

To study the effect of the structure charging on interlayer distance, the *ab initio* geometry optimisation was conducted for a series of structures with different total charges. To properly describe the charged structure, the multipole boundary conditions were imposed on the direction perpendicular to the surface. The results show that in both cases, the structure expands significantly while being charged. The effect of expansion is greater in the layers closer to the surface, leading to the delamination of an outer layer in the case of intercalation of the phosphorene, which is visible in Figure 8b, in the sudden rise of d_1_ interlayer distance after charge exceeds 8e. This suggests that interlayer covalent bonding is a crucial mechanism for achieving high specific capacities in phosphorene‐LIG heterostructures since van der Waals forces are too weak to provide sufficient layer stability at high structural charging.

ELF analysis confirmed covalent bonding within heterostructure layers and proper Li intercalation without bond formation. *Ab initio* geometry optimization revealed charging‐induced structural expansion with surface‐proximal layers experiencing pronounced effects, culminating in phosphorene outer layer delamination beyond 8e^−^ charge‐indicating interlayer covalent bonding's critical role in high‐capacity stability. These findings parallel Frison's ELF‐based bonding characterization methodology^[^ ^87^ ^]^ and complement Mansouri's first‐principles study of alkali metal behavior in G/P heterostructures.^[^ ^84^ ^]^ The heterogeneous architecture prevents graphene restacking, accommodates phosphorus' ≈300% lithiation volume change, facilitates rapid ion diffusion, and mitigates environmental degradation through passivation.

## 3 Conclusion

In summary, we have prepared a stable architecture containing nanoscale BP particle covalently bonded to a 3D graphene framework by P─C/P─O─C bond. By absorbing the laser energy, the BP particles are fragmented into nanoscale (below 100 nm) dimensions and simultaneously linked to the carbon network of laser‐processed graphene. Laser‐processing compatibility enables scalable manufacturing approaches for practical energy storage applications where laser photolise BP and polyimide film, which plays an essential role in the formation of stable P─C bonds and Pi–Pi sites, is confirmed in X‐ray absorption spectroscopy, and manifested in DFT. The BP‐LIG heterostructure‐based electrode exhibits a high reversible capacity of ≈860 mAh g^−1^ at 0.8 A g^−1^. The presence of stable covalent bonds (P─C and P─O─C) between nanoscale BP particles and the LIG network significantly improves structural stability, allowing the electrode to maintain consistent performance over prolonged cycling (100% CE over 700 cycles). This work demonstrates a single‐step lasing method for preparing high‐performance nanoscale BP particles for LIBs, which can extend to further rational design of BP anode in other alkali‐ion (Na/K ion) storage.

## 4 Experimental Section

### Materials

1‐M lithium hexafuorophosphate (LiPF_6_) in a mixture of ethylene carbonate (EC) and dimethyl carbonate (DMC) at the 1:1 volume ratio was purchased from Aldrich. The separator was made from glass microfiber GF/A (Whatman). Lithium foil (0.75 mm thick) was purchased from Aldrich.

### Few‐Layer Black Phosphorus Synthesis

Few‐layer black phosphorus (FLBP) was prepared by solvent‐assisted exfoliation of pre‐ground black phosphorus (BP, Smart Elements). To obtain the dispersion, 44 mg of BP was dispersed in 8 mL of deoxygenated 95% ethanol (Sigma Aldrich), deoxygenated by purging with high‐purity argon (Air Liquide). The dispersion was then sonicated using a horn probe ultrasonicator (Bandelin Sonopuls HD2200, 20 kHz) under a continuous argon stream, while maintaining the temperature between 0 and 3 °C using an ice‐cooled bath. Ultrasonication was carried out at a power of 40 W with a 0.5/0.5 s ON/OFF cycle for 4 h to break the van der Waals bonds within the BP crystal. The resulting suspension contained FLBP flakes with an average size of ≈280 nm, with the majority of flakes ranging from 100 nm to 1 µm. The suspension was subsequently used for drop casting onto polyimide foil. The detailed synthesis procedure for FLBP followed the method described in the literature.^[^ ^88^ ^]^

The particle size distribution of FLBP in ethanol was determined by dynamic light scattering (DLS) using a Zetasizer Nano ZS particle analyzer (Malvern Panalytical, UK) equipped with a 633 nm laser. The analyzer measures particle sizes ranging from 0.3 nm to 10 µm. FLBP suspension in ethanol was analyzed in the backscatter configuration (173° scattering angle), with measurements repeated five times (Figure S6, Supporting Information).

### X‐ray Absorption Spectroscopy

The high‐resolution C1s X‐ray absorption spectra of LIG and BP‐LIG samples were recorded using the facilities of the HE‐SGM beamline (HE‐SGM) at the BESSY II synchrotron radiation source of Helmholtz–Zentrum Berlin (HZB). The spectra were acquired under ultra‐high vacuum conditions (P ≈ 1 × 10^−9^ Torr) at room temperature. The NEXAFS spectra were obtained by recording the total electron yield (TEY) using the PEY/TEY detector. The monochromator energy resolution near the C1s absorption edge (hv ≈285 eV) was ≈100 meV. The size of the X‐ray spot on the samples was hor/ver ≈1200 × 200 µm. The photon energies in the range of the fine structure of the C1s X‐ray absorption spectra were calibrated against the energy position of the π‐resonance in the C1s X‐ray absorption spectrum of HOPG (hv ≈ 285.45 eV).^[^ ^89^ ^]^ No radiation damages to the LIG and BP‐LIG were observed during the spectra acquisition.

### X‐ray Photoelectron Spectroscopy

The survey, HR C1s, and HR P2p photoelectron spectra were acquired at hν = 730 eV using an electron energy analyser Scienta R3000 (Scienta). The analyzer pass energy was set to 200 and 50 eV, respectively. The C1s, Au4f, and valence band spectra of the reference HOPG and Au samples were recorded to calibrate the analyser work function. The detection angle was close to that of normal emission. To analyze the data, the UNIFIT software was used for the spectra fitting by the Gaussian/Lorentzian convolution functions.^[^ ^90^, ^91^ ^]^ The optimisation of the background parameters was done simultaneously during the deconvolution procedure.

### Electrochemical Measurements

The LIBs were fabricated in a Swagelok cell arrangement in an Ar‐filled glove box. Where BP‐LIG was used as the anode, lithium hexafluorophosphate as the electrolyte, Whatman glass fiber as the separator, and Li foil as the cathode. After assembly, the Swagelok cells were sealed and waited for 12 h. to stabilize the open circuit potential before being used for electrochemical testing. Our anode (BP‐LIG) has an approximate area of ≈0.78 cm^2^, resulting in ≈0.1 mg of active material.

### Computational Details

The DFT calculations were conducted using the Quantum ATK package.^[^ ^92^ ^]^ The geometry of each model was optimized using L‐BFGS geometry optimizer until forces were below 0.05 eV Å^−1^. The interatomic forces were calculated using the Linear Combination of Atomic Orbitals (LCAO) method as implemented in Quantum ATK.^[^ ^93^ ^]^ The form of the functional used was chosen to be Perdew–Burke–Ernzerhof (PBE). The atomic basis set from the Pseudo Dojo database was used.^[^ ^94^ ^]^ The k‐space was uniformly sampled with a density equal to 7 Å. The energy cutoff for the mesh was set to 100 Hartree. The van der Waals interactions were described by the introduction of GrimmeDFTD3 dispersion correction.^[^ ^95^ ^]^

## Conflict of Interest

The authors declare no conflict of interest.

## Supporting information



Supporting Information

## Data Availability

The data that support the findings of this study are available from the corresponding author upon reasonable request.
