# Supplementary material for: Laser‐Induced Heterostructuring of Graphene Passivated Nanoscale Black Phosphorus Frameworks for Lithium‐Ion Battery Anodes
Source: Small. 2025 Sep 1;21(41):e04480. doi: 10.1002/smll.202504480 (PMC12530024; doi:10.1002/smll.202504480)
Supplement: Supplementary file 1 — Supporting Information [file SMLL-21-e04480-s001.docx]

**Laser-Induced Heterostructuring of Graphene Passivated Nanoscale Black Phosphorus Frameworks for Lithium-Ion Battery Anodes**

*Sujit Deshmukh,* Pawel Jakobczyk, Krzysztof Pyrchla, Maria Brzhezinskaya, Mateusz Ficek, Bing Yang,* *Nianjun Yang,* Robert Bogdanowicz**

Dr. S. Deshmukh, K. Pyrchla, Dr. P. Jakobczyk, Dr. M. Ficek, Prof. R. Bogdanowicz

Department of Metrology and Optoelectronics, Faculty of Electronics, Telecommunications, and Informatics, Gdansk University of Technology, 11/12 G. Narutowicza Str., 80-233 Gdansk, Poland

Dr. habil. M. Brzhezinskaya

Helmholtz-Zentrum Berlin für Materialien und Energie, Hahn-Meitner-Platz 1, 14109 Berlin, Germany

Dr. B. Yang

Shenyang National Laboratory for Materials Science, Institute of Metal Research (IMR), Chinese Academy of Sciences (CAS), No. 72 Wenhua Road, Shenyang 110016, China.

Prof. N. Yang

Department of Chemistry, Hasselt University, Agoralaan-Gebouw F, Wetenschapstoren Kantoor F4.12, 3590 Diepenbeek, Belgium.

Institute of Materials Research, Hasselt University, Wetenschapspark 1, 3590 Diepenbeek, Belgium.


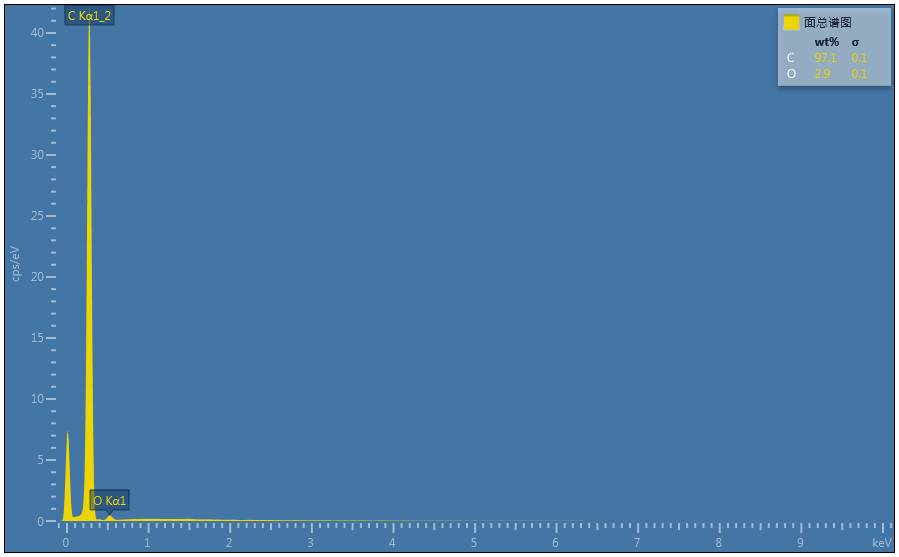


**Figure S1.** Energy-dispersive X-ray spectroscopy spectrum of LIG.


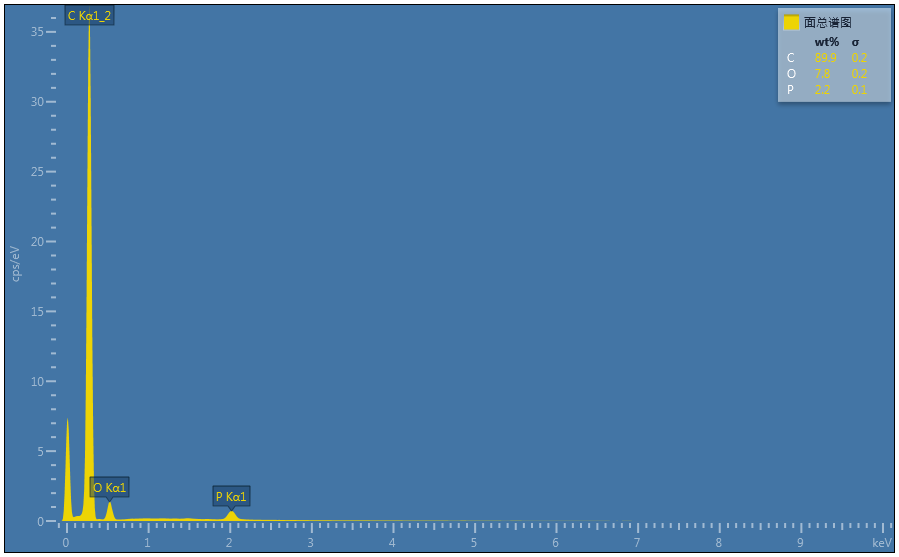


**Figure S2.** Energy-dispersive X-ray spectroscopy spectrum of BP-LIG.


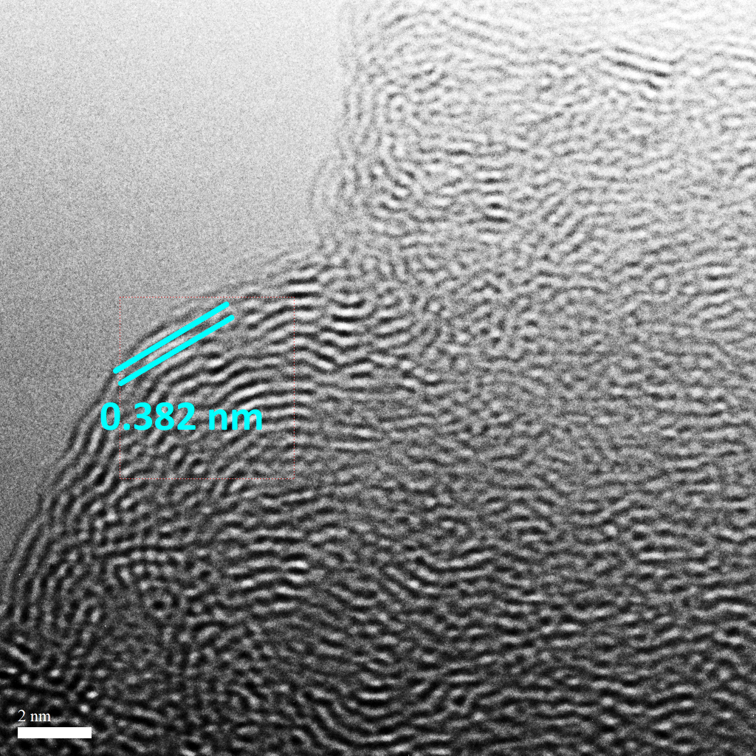


**Figure S3.** HRTEM image of BP-LIG sample.


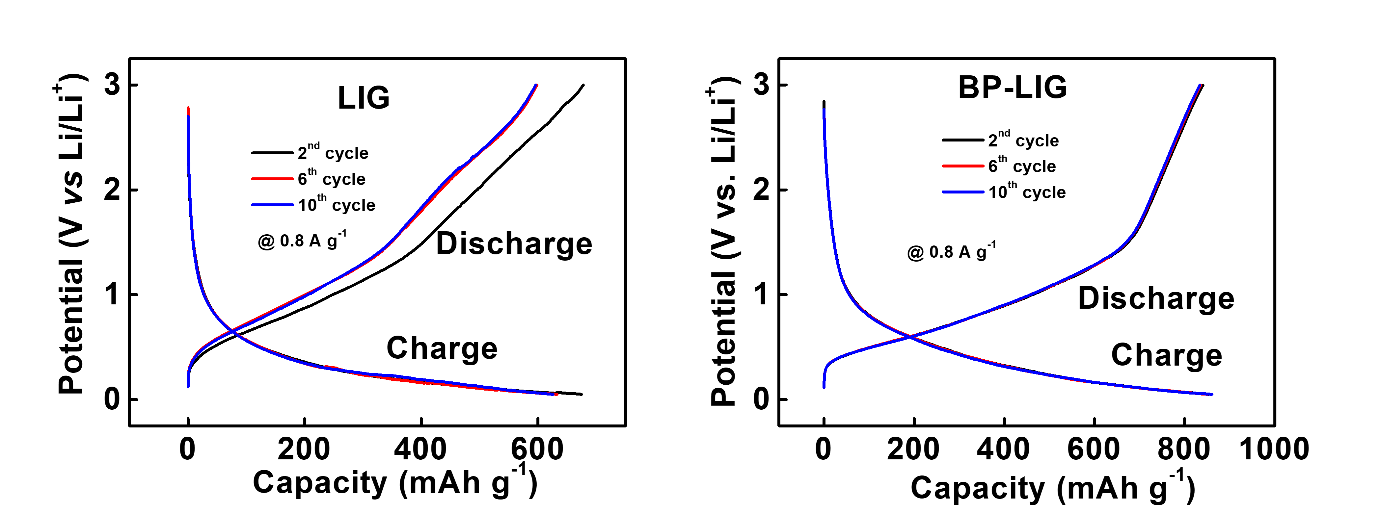


**Figure S4.** Voltage profile of LIG and BP-LIG hybrid recorded at 2^nd^, 6^th^ and 10^th^ cycles.

**Figure S5.** Reproducibility test of three independently prepared electrodes of LIG and BP-LIG upto 100 GCD cycles at 2 A g^-1^.

**Figure S6.** The particle size distribution of few-layer black phosphorus in ethanol.
